# Primary neuroendocrine small cell carcinoma of the parotid gland: A case report and review of the literature

**DOI:** 10.3892/ol.2014.2258

**Published:** 2014-06-17

**Authors:** MINDA LIU, MING ZHONG, CHANGFU SUN

**Affiliations:** 1 Department of Oromaxillofacial-Head and Neck Surgery, School of Stomatology, China Medical University, Shenyang, Liaoning 110002, P.R. China; 2 Department of Oral Pathology, Central Laboratory, School of Stomatology, China Medical University, Shenyang, Liaoning 110002, P.R. China

**Keywords:** neuroendocrine small cell carcinoma, immunohistochemistry, parotid gland

## Abstract

Small cell carcinoma (SCC) is a malignant epithelial tumor that predominantly arises in the lungs. Primary SCC of the parotid gland is rare and difficult to diagnose by analysis of frozen sections obtained during surgery. Due to the aggressive nature of SCC and the frequent occurrence of distant metastases, identification of the disease is important. The current study reports the case of a male patient who presented with a right parotid gland mass. The tumor was resected and evaluated by light microscopy and immunohistochemical analysis. Immunohistochemically, the tumor was positive for cytokeratin, epithelial membrane antigen, cluster of differentiation 117, synaptophysin and thyroid transcription factor-1, which indicated that the tumor was a SCC of the parotid gland. An extended resection of the right parotid gland mass and dissection of the facial nerve were performed. Following discharge from the hospital, the patient received radiation therapy postoperatively. The patient has remained disease free during five months of follow-up.

## Introduction

Small cell carcinoma (SCC) was initially identified in the lung as oat cell sarcoma (1,2). The first case of SCC of the head and neck was reported by Olofsson and Van Nostrand in 1972 (3), and was found in the larynx. SCC was once considered to be a type of undifferentiated carcinoma, however, it has now been distinguished from undifferentiated carcinoma and can be divided into two subtypes, neuroendocrine and ductal. Neuroendocrine SCC often occurs in the nasal cavity, whereas primary neuroendocrine SCC of the parotid gland is extremely rare, accounting for <1% of all tumors of the salivary gland (4). Primary neuroendocrine SCCs of the parotid gland are known to be aggressive, always presenting with early distant metastasis and frequently exhibiting recurrence (5). Due to the extreme rarity of these cases, no definite treatment regimen can be followed. The definitive diagnosis relies on immunohistochemistry, and currently, the treatment regimen includes surgery, radiotherapy and chemotherapy, or a combination of these methods (6). In the present study, clinical, cytological and immunophenotypic features of primary neuroendocrine SCC in parotid gland are discussed. Patient provided written informed consent.

## Case report

**Figure 1:**
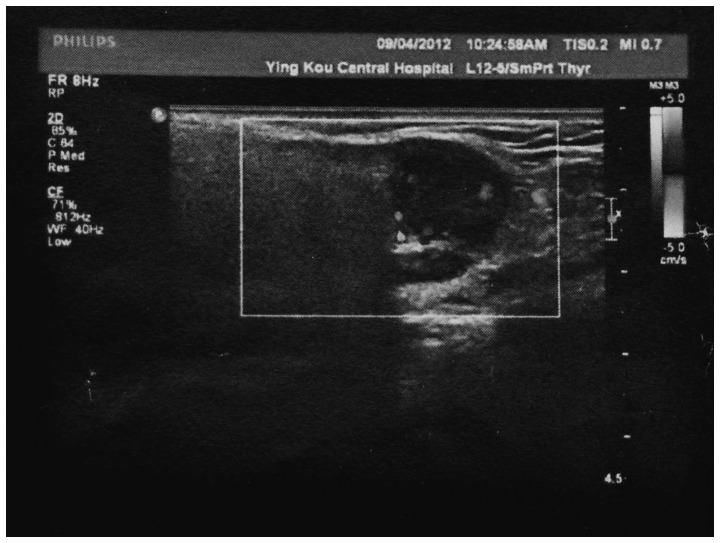
Ultrasonography of the parotid gland.

A 59-year-old male consulted to the Department of Oromaxillofacial-Head and Neck Surgery, School of Stomatology, China Medical University (Shenyang, China) due to a painless mass in the right parotid that had been progressively enlarging for two months. Upon admission, facial nerve palsy was not observed, and the mass appeared to be nodular. Ultrasonography revealed a 2.2×1.5-cm well-defined hypoechoic lesion in the right parotid gland (Fig. 1). A computed tomography (CT) scan of the thorax and ultrasonography of the thyroid gland did not reveal any involvement of other sites. An ‘S’ incision was made from the front of the earlobe to the jaw, separate and protecting the mandible branch and cervical branch of the facial nerve. The tumor was located behind and below the cervical branch of the facial nerve. Adhesions were located between the tumor and great auricular nerve, additionally enlarged lymph nodes were located beside the tumor. An extended resection of the right parotid gland mass, excision of the great auricular nerve and dissection of the facial nerve were subsequently performed. Following surgery, the patient received post-operative radiation therapy and remained disease-free during five months of follow-up.

A mass of 1.7×2.4×1.4 cm^3^ in size was resected to obtain an intraoperative frozen section. The margins of the mass were well defined, the texture of the section was solid and the coloration was pink and white. The intraoperative frozen section revealed that the mass was an epithelial malignant tumor. Such a result indicated that the tumor should be evaluated by immunohistochemistry. The results of the immunohistochemical analysis indicated that the tumor was a neuroendocrine SCC.

**Figure 2:**
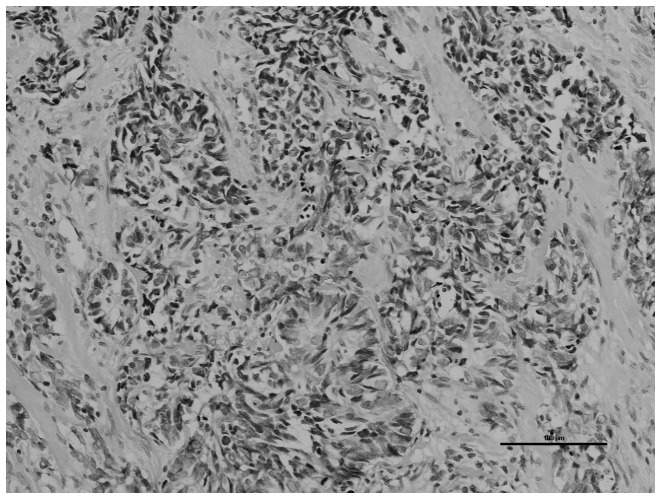
Hematoxylin and eosin-stained small cell carcinoma (SSC) of the parotid gland (scale bar, 100 μm; magnification, ×200).

Under a light microscope, the tumor cells stained with hematoxylin and eosin were shown to be arranged into irregular nests. Tumor cells were observed palisading around the cell nest with a moderate amount of fibrous mesenchyme. The tumor cells were small with poorly-defined borders, bare nuclei and sparse cytoplasm. The nuclei were round, oval or partially fusiform. The chromatin was fine and granular and the nucleoli were not evident. A section of the nuclei was twisted and fragmental necrosis was visible (Fig. 2).

**Figure 3:**
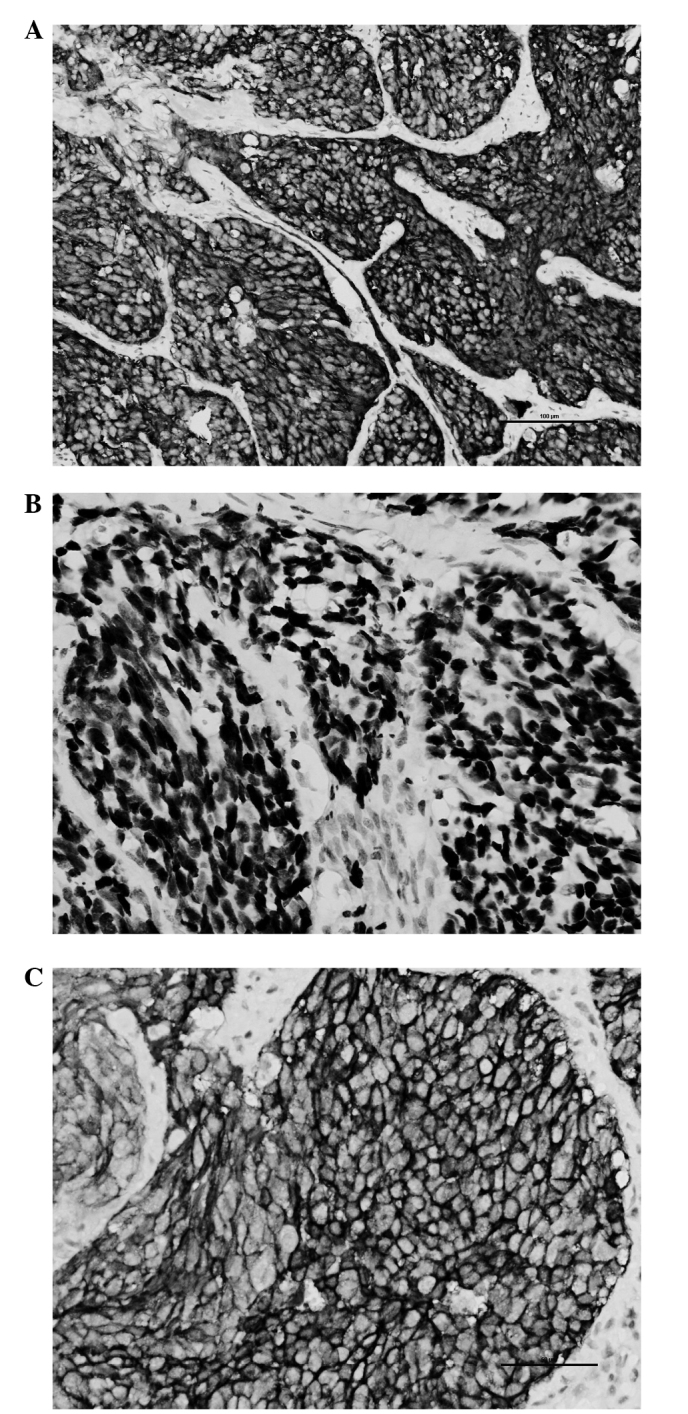
Immunohistochemical staining analysis indicating a carcinoma positive for (A) synapsin (SYN), (B) thyroid transcription factor-1 (TTF-1) and (C) cluster of differentiation 117 (CD117) (scale bar, 100 μm ; magnification, ×200).

The immunohistochemical study demonstrated that the tumor cells were positive for cytokeratin (CK), epithelial membrane antigen (EMA), cluster of differentiation (CD)117, synaptophysin (SYN) and thyroid transcription factor (TTF)-1. SYN, TTF-1 and CD117 are all known neuroendocrine cell markers (Fig. 3A–C). SYN is a specific marker of nerve and epithelial tumors and is often used as a marker for neuroendocrine tumors (7). TTF-1 is frequently expressed in the epithelial cells of the thyroid glands and lungs, and the majority of lung SCCs and atypical neuroendocrine tumors are also immunohistochemically positive for TTF-1 (8). CD117 is predominantly expressed in gastrointestinal stromal tumors, but may also be expressed in SCC of the lung (9). A diagnosis of SCC can be determined if a tumor is positive for at least one of these neuroendocrine cell markers.

Immunohistochemically, CD117 may also be expressed in SCC of the lung, and TTF-1 may be expressed in carcinomas of the thyroid glands or lungs. Therefore, according to the immunohistochemical results, it was deemed likely that the tumor in the present study was a metastatic carcinoma.

Overall, by considering the medical history, histopathological findings, immunohistochemical analysis, CT scan results and ultrasonography of the thyroid gland, the tumor was not considered to be a metastatic carcinoma. A final diagnosis of primary neuroendocrine SCC was reached.

## Discussion

SCC is a highly aggressive epithelial malignancy that often occurs in the lungs, and extrapulmonary SCC is identified in only 2.5–5% of cases (10). In cases affecting the head and neck, SCC often occurs in the larynx, nasal cavity, paranasal sinuses, pharynx, oral cavity, cervical esophagus and salivary glands (11–13). SCC in the salivary glands is rare, comprising <1% of all tumors that occur in the salivary glands. Generally, primary SCC of the parotid gland clinically presents as a painless, fast-growing mass that develops in three to six months. The tumor is often observed in patients between 50 and 80 years old and is more frequently identified in males. The largest reported series of SCC of the major salivary glands (n=15) revealed a 73% male predominance (14).

In SCC, the cells are 2–3 times larger than the mature small lymphocytes and are round or oval in shape, consisting of solid sheets and nest of tumor cells. The carcinoma is also positive for neuroendocrine markers. In the majority of SCC cases, the tumor cells express at least one of the neuroendocrine markers (15). Huntrakoon (16) reported that membrane-bound, dense-core neurosecretory granules were observed in ~20% of cases with SCC of the salivary glands. Eversole and Knapp (17) demonstrated that 47% of SCC tumors contained neuroendocrine granules with diameters ranging between 80 and 240 nm. The expression of CK, EMA, CD117, SYN and TTF-1 in the present case indicated that the carcinoma was a neuroendocrine SCC.

The differential diagnosis includes primary neuroendocrine SCC or metastatic tumors. A variety of tumors can be considered for the differential diagnosis, including primary epithelial ductal tumors, terminal duct carcinoma, poorly-differentiated adenoid-cystic carcinoma, basal cell adenoma or carcinoma, metastatic lesions and Merkel cell carcinoma of the skin or lungs (11,18). The definitive diagnosis of neuroendocrine SCC should be made by immunohistochemical analysis. Histopathological examination alone is not sufficient to determine a diagnosis for primary SCC of the parotid gland. Medical history and image-based studies must also be performed to exclude metastatic SCC.

To the best of our knowledge, primary SCC of the parotid gland has an improved prognosis compared with other sites, however, local recurrence and distant metastases have been reported to occur in >50% of patients following the diagnosis. The five-year survival rate of patients with tumors arising in the major salivary glands ranges between 13 and 46%, while the two-year survival rate is 70% (18). SCC of the head and neck is also known for its hematogenous dissemination (5). A study by the University of Virginia reported an incidence of distant spread of 71% (5), whereas the University of Miami reported an incidence of distant spread of 25% (5). The present report demonstrated that SCC occurring in the parotid gland has an improved prognosis compared with SCC arising in the lung.

The size of the primary neuroendocrine SCC is considered to be the most significant prognostic factor. A previous study reported that tumors with a diameter of >3 cm have a poorer outcome than smaller tumors (18). In the study, the largest tumor diameter measured by ultrasonography was 2.1 cm. An additional prognostic factor is the type and number of neuroendocrine markers. Tumors that expressed more than four different neuroendocrine markers exhibited an improved outcome compared with those that expressed only two or three neuroendocrine markers (18). Another study reported that the Kaplan-Meier estimate of the proportion of patients with SCC of the head and neck who survived for one and two years was 63 and 26%, respectively. Furthermore, the proportion of patients with SCC of the head and neck who remained disease-free at one and two years was 71 and 44%, respectively (5).

Due to the rarity of primary neuroendocrine SCC, there is currently no definite treatment regimen, and there is little prognostic data on neuroendocrine SCC of the parotid gland. At present, the majority of cases of neuroendocrine SCC of the parotid gland are treated by surgery and radiation therapy (4,5,14,18–21). Surgical treatment always comprises removal of the lesions, an expanded resection and an ipsilateral modified neck dissection. In total, 75% of local recurrence occurs in cases that have only been treated by surgery; with the combination of surgery and radiotherapy, the recurrence rate is reduced to 20% (18). In addition, the three-year survival rate of patients undergoing treatment is 25% with surgery alone and 80% when combining surgery with radiotherapy (12). Jorcano *et al* (18) reported a case of primary SCC of the parotid gland that was treated only by radiotherapy and reported a three-year survival outcome. In conclusion, it is recommended that surgery be combined with radiotherapy as a treatment for primary neuroendocrine SCC. The study aimed to highlight the possibility of neuroendocrine SCC of the parotid gland as a differential diagnosis when the tumor is difficult to diagnose.
